# Acute Fibrinous and Organizing Pneumonia Versus Organizing Pneumonia: A Comparative Study of Clinical, Radiological, Histological, and Immunophenotypic Features

**DOI:** 10.3390/jcm15186998

**Published:** 2026-09-10

**Authors:** Xuexue Wu, Xiaoyuan Li, Mengqian Li, Zuoyu Liang, Ping Zhou, Chan Yang, Lingping Xie, Lili Jiang

**Affiliations:** 1 Department of Pathology, West China Hospital, Sichuan University, Chengdu 610041, China; wuxxdoct@163.com (X.W.); lixiaoyuanxh@163.com (X.L.); limengqian@wchscu.edu.cn (M.L.); zuoyu_liang@hotmail.com (Z.L.); zhouping90@163.com (P.Z.); chanyang6@hotmail.com (C.Y.); xielingping99@163.com (L.X.); 2 Department of Pathology, West China Tianfu Hospital, Sichuan University, Chengdu 610213, China

**Keywords:** acute fibrinous and organizing pneumonia, organizing pneumonia, immunohistochemistry, macrophage, C-reactive protein, differential diagnosis

## Abstract

**Background**: The relationship between acute fibrinous and organizing pneumonia (AFOP) and organizing pneumonia (OP) remains debated, and direct comparative evidence is limited. We aimed to delineate their differences across clinical, radiological, histological, and immunophenotypic dimensions. **Methods**: We conducted a retrospective study of 85 consecutive patients with pathologically confirmed AFOP (*n* = 49) or OP (*n* = 36). Clinical data, radiological patterns, laboratory findings, and immunohistochemical profiles of alveolar epithelial, macrophage, lymphocyte, vascular, and mesenchymal markers were comprehensively compared. **Results**: The two groups were comparable in demographics, clinical symptoms, and dominant radiological patterns. However, median C-reactive protein (CRP; 80.45 vs. 12.65 mg/L, *p* < 0.001) and neutrophil-to-lymphocyte ratio (NLR; 4.56 vs. 2.42, *p* = 0.013) were higher in AFOP, with CRP demonstrating an apparent area under the curve (AUC) of 0.798 for discriminating AFOP from OP in this derivation cohort. After Benjamini–Hochberg correction for multiple comparisons, CD68 was significantly higher, and CD38 was significantly lower in AFOP than in OP. Trends were also observed for CD163, surfactant protein A (SP-A), and CD34, but these did not remain statistically significant after correction. All three deaths and all cases requiring mechanical ventilation occurred in the AFOP group, though these differences were not statistically significant. **Conclusions**: Despite clinical and radiological overlap, AFOP and OP exhibit distinct systemic inflammatory responses and pulmonary immunophenotypes, with AFOP characterized by macrophage-predominant inflammation and qualitatively fewer CD34+ microvessels within fibrin balls. These immunophenotypic findings are descriptive and require further validation.

## 1. Introduction

Organizing pneumonia (OP) is a non-specific pulmonary response to lung injury, histologically defined by polypoid granulation tissue (Masson bodies) in distal airspaces, with largely preserved lung architecture [1,2]. Although OP generally carries a favorable prognosis and responds well to corticosteroids, its clinical and radiological presentations are highly heterogeneous, often mimicking infectious pneumonia, interstitial lung disease, or malignancy [3].

In 2002, Beasley et al. first described acute fibrinous and organizing pneumonia (AFOP) as a distinct histologic pattern of acute lung injury characterized by prominent intra-alveolar fibrin deposition, typically manifesting as “fibrin balls” [4]. Subsequently, the American Thoracic Society/European Respiratory Society (ATS/ERS) recognized it as a rare histological pattern within the spectrum of idiopathic interstitial pneumonias in 2013 [5]. Since then, AFOP has been increasingly recognized as an uncommon but important histologic pattern that can occur idiopathically or be secondary to a wide range of conditions, including connective tissue diseases, drug reactions, infections, and transplantation [6,7,8].

AFOP shares similarities with OP in clinical manifestations, radiological characteristics, and histological features. Therefore, some studies have proposed that AFOP may represent a pattern within the OP spectrum [2,9] or even an early more acute phase of OP [10]. However, direct comparative evidence between AFOP and OP remains limited. As a result, whether AFOP represents a truly distinct clinical entity or a variant within the OP spectrum remains a subject of ongoing debate.

Given the prominent intra-alveolar fibrin in AFOP and prior evidence linking fibrin deposition to elevated C-reactive protein (CRP) [10,11], we focused on CRP as a marker of acute-phase inflammation. In addition, the neutrophil-to-lymphocyte ratio (NLR) is a routinely available marker of neutrophil–lymphocyte imbalance and has been associated with severity and poor outcomes in acute lung injury and severe respiratory infections [12]. Therefore, we also evaluated NLR as a candidate marker of innate immune predominance. In this study, we report the largest cohort to date comparing AFOP and OP. We aimed to delineate the differences between these two conditions by comprehensively analyzing their clinical, radiological, histological, and immunophenotypic features.

## 2. Materials and Methods

### 2.1. Study Design and Patients

We conducted a retrospective study of 85 consecutive patients with pathologically confirmed AFOP or OP at the West China Hospital of Sichuan University between January 2019 and December 2020. Patients were classified into the AFOP group or the OP group according to the final pathological diagnosis. All lung biopsy specimens were independently reviewed by two experienced pathologists according to predefined diagnostic criteria (see Table A1). Lung tissue specimens were mainly obtained via percutaneous lung biopsy (PLB) and transbronchial lung biopsy (TBLB). To minimize sampling errors due to spatial lesion heterogeneity, diagnoses of AFOP or OP were confirmed solely upon the clear presence of typical morphological features via consensus review.

Detailed clinical data, including the clinical features, radiological characteristics, laboratory tests, treatment regimens, and clinical outcomes, were collected from the electronic medical records. Follow-up data were supplemented by outpatient records and telephone interviews through December 2025. All data were verified by double data entry.

**Figure A1:**
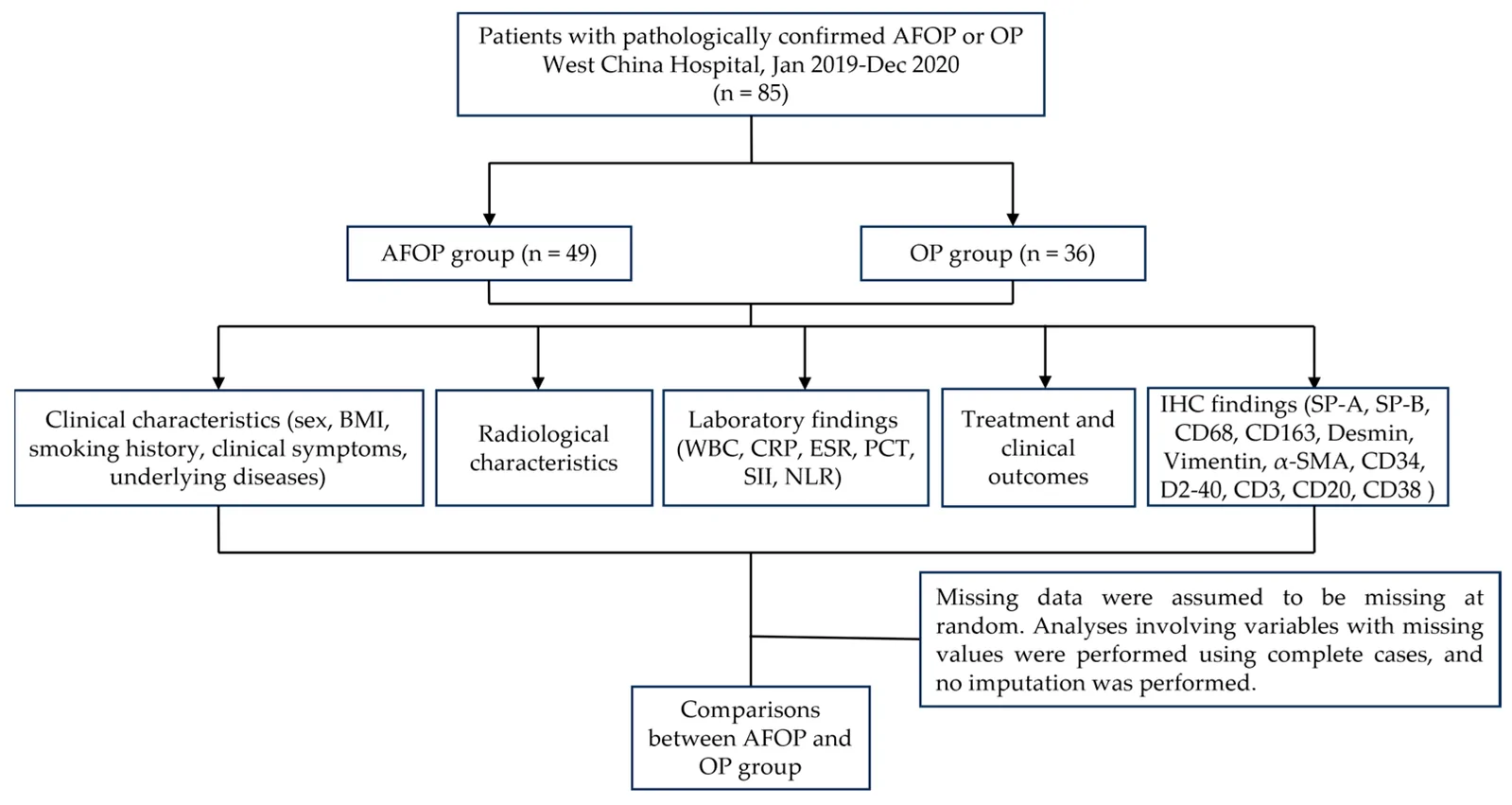
STROBE flow diagram of patient inclusion and analysis subsets with missing data.

The study followed the STROBE reporting guidelines. Missing data were assumed to be missing at random, and analyses involving variables with missing values were performed using complete cases without imputation. The patient flow and analysis subsets are shown in Figure A1. No prospective sample size calculation was performed; this was an exploratory retrospective study.

This study was approved by the Biomedical Ethics Review Committee of West China Hospital, Sichuan University (Approval No. 739), and the requirement for informed consent was waived.

### 2.2. HE and Immunostaining

Formalin-fixed paraffin-embedded (FFPE) tissue sections (4 µm) were stained with hematoxylin and eosin (H&E) and subjected to immunohistochemistry (IHC). IHC was performed on Leica Bond-Max (Leica Biosystems, Wetzlar, Germany) or Roche Ventana platforms (Ventana Medical Systems, Inc., Tucson, AZ, USA) using the EnVision two-step method (Dako, Glostrup, Denmark). The following antibodies were used: surfactant protein A (SP-A, clone 6F10, Gene Tech (Shanghai) Co., Ltd., Shanghai, China), surfactant protein B (SP-B, clone 1B9, ZSGB-BIO, Beijing, China), CD34 (clone EP88, ZSGB-BIO, Beijing, China), D2-40 (clone D2-40, ZSGB-BIO, Beijing, China), desmin (clone MX046, Maixin Biotech, Fuzhou, China), α-smooth muscle actin (α-SMA, clone UMAB237, ZSGB-BIO, Beijing, China), vimentin (clone UMAB159, ZSGB-BIO, Beijing, China), CD68 (clone PG-M1, ZSGB-BIO, Beijing, China), CD163 (clone 10D6, ZSGB-BIO, Beijing, China), CD3 (clone LN10, ZSGB-BIO, Beijing, China), CD20 (clone L26, ROCHE, Ventana Medical Systems, Tucson, AZ, USA), and CD38 (clone MX044, ZSGB-BIO, Beijing, China). Detailed information on the antibodies is provided in Table A2.

Immunoreactivity was quantified using a semi-quantitative immunoreactivity score (IRS). The IRS was calculated by multiplying the staining intensity (0–3) by the percentage of positively stained cells (0–4). The staining intensity was scored as 0 (negative, no staining), 1 (weak, light yellow), 2 (moderate, brown yellow), or 3 (strong, dark brown). The percentage of positive cells was scored as 0 (<5%), 1 (5–25%), 2 (26–50%), 3 (51–75%), or 4 (>75%). All assessments were performed independently by two experienced pathologists. Any discrepancies were resolved by consensus or by a third pathologist.

Interobserver agreement for the histological classification of AFOP versus OP was assessed using Cohen’s kappa. For semi-quantitative IRS scores, weighted kappa (quadratic weights) was calculated for each marker. Markers with zero variance in both raters were recorded as having 100% concordance; kappa could not be calculated for these markers. Agreement levels were interpreted according to Landis and Koch [13].

### 2.3. Statistical Analysis

Quantitative data were expressed as the mean ± SD and compared by the independent samples *t*-test when normally distributed; otherwise, the median (interquartile range) and the Mann–Whitney U test were used. Normality and homogeneity of variance were assessed using the Shapiro–Wilk test and Levene’s test, respectively. Categorical variables were compared by the chi-square test or Fisher’s exact test. Effect sizes for non-parametric comparisons were reported as Cliff’s delta, with Hodges–Lehmann median differences and 95% CIs (confidence intervals) for continuous variables. For Cliff’s delta, positive values indicate higher scores in the AFOP group relative to the OP group, and negative values indicate lower scores in the AFOP group. The Benjamini–Hochberg (BH) procedure was applied to control the false discovery rate across the 12 IHC markers. Receiver operating characteristic (ROC) curves and optimal cut-offs (maximizing the Youden index) were generated for CRP and NLR; sensitivity, specificity, positive predictive value (PPV), negative predictive value (NPV), and likelihood ratios (LR) were calculated at the optimal cut-offs. Bootstrap internal validation was performed with 2000 stratified resamples. The optimism-corrected AUC was calculated as the apparent AUC minus the mean optimism (mean bootstrap AUC minus apparent AUC). Independent associations with AFOP were assessed by univariable and multivariable Firth penalized logistic regression adjusted for age and cardiovascular disease (CRP per 10 mg/L). A sensitivity model additionally included NLR. For rare outcomes (death, mechanical ventilation), the post hoc power was estimated by simulation (10,000 replicates) at α = 0.05. Effect size calculations, ROC analyses, bootstrap internal validation, BH correction, and post hoc power simulations were performed in R (version 4.5.0). All other statistical analyses were conducted with IBM SPSS Statistics version 26.0. A two-sided *p* value < 0.05 was considered statistically significant.

## 3. Results

### 3.1. Clinical Characteristics

A total of 85 patients were enrolled, including 49 with AFOP and 36 with OP. The baseline demographic and clinical characteristics of the two groups are summarized in Table 1. The mean age was 54.34 ± 15.15 years in the AFOP group and 53.76 ± 14.82 years in the OP group, with no significant difference (*p* = 0.318). The sex distribution, body mass index (BMI), and smoking history were also comparable between the two groups (all *p* > 0.05).

The most common clinical symptoms in both groups were cough (65.31% vs. 66.67%), sputum production (38.78% vs. 47.22%), fever (28.57% vs. 41.67%), chest pain (28.57% vs. 19.44%), and dyspnea (16.33% vs. 13.89%), with no significant differences between groups (all *p* > 0.05).

Regarding underlying diseases, cardiovascular disease was significantly more prevalent in the AFOP group than in the OP group (22.45% vs. 3.03%, *p* = 0.023). The prevalence of other comorbidities, including respiratory diseases, neoplastic diseases, hematological diseases, metabolic diseases, digestive diseases, urinary system diseases, and autoimmune diseases, did not differ significantly between the two groups (all *p* > 0.05).

Regarding the biopsy approaches, PLB was performed in 47/49 (95.92%) patients in the AFOP group and 30/36 (83.33%) patients in the OP group, while the remaining patients underwent TBLB. The difference between the two groups was not statistically significant (*p* = 0.156).

### 3.2. Radiological Characteristics

We applied the CT classification of Kim et al. [14], which divides AFOP into ground-glass opacity (GGO)/consolidation-dominant, nodule-dominant, and fibrosis-dominant patterns. The radiological characteristics of the two groups are summarized in Table 2.

Regarding the dominant pattern, both groups most frequently presented with a nodule-dominant pattern (51.02% in AFOP vs. 47.22% in OP), followed by a GGO/consolidation-dominant pattern (38.78% vs. 36.11%) and a fibrosis-dominant pattern (10.20% vs. 16.67%), with no significant difference between groups (*p* = 0.712). The anatomical location of lesions also did not differ significantly between groups (*p* = 0.275), with a diffuse or random distribution being the most common in both groups.

Specific radiological findings were likewise largely comparable between the two groups. Nodules or masses were the most frequent finding, followed by fibrosis, consolidation, and ground-glass opacities. Pleural effusion, lymphadenopathy, and air bronchograms also occurred at similar frequencies (all *p* > 0.05). The reverse halo sign was observed in only one OP patient and was absent in the AFOP group.

### 3.3. Laboratory Findings

The laboratory findings of the two groups are summarized in Table 3. Laboratory markers were not available in all patients; CRP was available in 65/85 patients, with NLR in 83/85 patients, and ROC analyses were based on these marker-specific complete-case cohorts. Compared with the OP group, the AFOP group had significantly higher CRP (80.45 mg/L vs. 12.65 mg/L, *p* < 0.001) and NLR (4.56 vs. 2.42, *p* = 0.013). The difference in CRP corresponded to a large effect size (Cliff’s delta = 0.60, 95% CI: 0.32–0.78; Hodges–Lehmann median difference = 43.8 mg/L, 95% CI: 17.5–73.8). NLR showed a medium effect size (Cliff’s delta = 0.32, 95% CI: 0.07–0.54; Hodges–Lehmann median difference = 1.32, 95% CI: 0.31–2.54). The white blood cell count (WBC), erythrocyte sedimentation rate (ESR), procalcitonin (PCT), and systemic immune-inflammation index (SII) did not differ significantly between the two groups (all *p* > 0.05).

**Figure 1:**
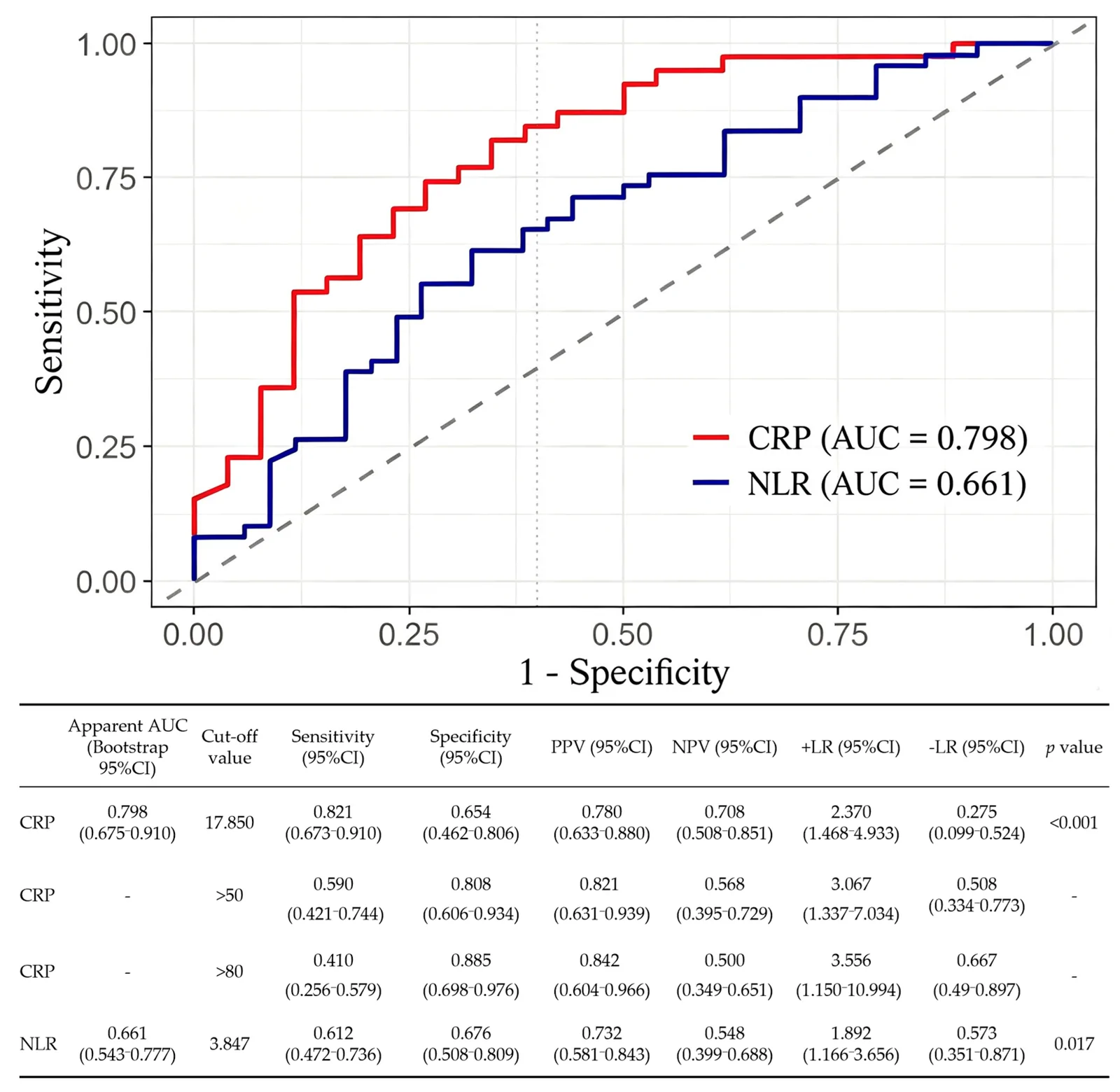
ROC curves of CRP and NLR for discriminating AFOP from OP. Apparent AUCs: CRP 0.798 (bootstrap 95% CI: 0.675–0.910), NLR 0.661 (bootstrap 95% CI: 0.543–0.777); optimism-corrected AUCs were 0.798 and 0.660, respectively. The table provides diagnostic performance at the Youden-derived optimal cut-offs and at two higher clinically relevant CRP thresholds. PPV/NPV reflect the AFOP prevalence in each marker-specific cohort: CRP cohort, 39/65, 60.0%; NLR cohort, 49/83, 59.0%. *p* values refer to DeLong’s test for the ROC curves; no *p* values are reported for the higher CRP thresholds because they were selected post hoc for descriptive purposes. The dashed diagonal line represents the reference line (AUC = 0.5). ROC: receiver operating characteristic; CRP: C-reactive protein; NLR: neutrophil-to-lymphocyte ratio; AFOP: acute fibrinous and organizing pneumonia; OP: organizing pneumonia; AUC: area under the curve; CI: confidence interval; PPV: positive predictive value; NPV: negative predictive value; +LR: positive likelihood ratio; −LR: negative likelihood ratio.

Given these differences, the discriminatory performance of CRP and NLR for distinguishing AFOP from OP was further evaluated by ROC analysis (Figure 1). For CRP, the apparent AUC was 0.798 (bootstrap 95% CI: 0.675–0.910; *p* < 0.001) and the optimism-corrected AUC was 0.798 after bootstrap internal validation with 2000 resamples. For NLR, the apparent AUC was 0.661 (bootstrap 95% CI: 0.543–0.777; *p* = 0.017), with an optimism-corrected AUC of 0.660. At the optimal cut-off (17.85 mg/L), CRP showed a sensitivity of 82.1% (95% CI: 67.3–91.0) and specificity of 65.4% (95% CI: 46.2–80.6), with a positive likelihood ratio of 2.37 and negative likelihood ratio of 0.275. NLR had a sensitivity of 61.2% (95% CI: 47.2–73.6), specificity of 67.6% (95% CI: 50.8–80.9), positive likelihood ratio of 1.89, and negative likelihood ratio of 0.57 at a cut-off of 3.85. At the optimal cut-off, CRP had a PPV of 78.0% and NPV of 70.8%; NLR had a PPV of 73.2% and NPV of 54.8%. The complete metrics are shown in Figure 1. These AUC estimates are from a derivation cohort and require external validation in independent cohorts.

Because the Youden-derived optimal CRP cut-off was only mildly elevated and may have limited clinical specificity, we additionally evaluated two higher thresholds. At a CRP threshold of >50 mg/L, the sensitivity was 59.0% (95% CI: 42.1–74.4%), specificity 80.8% (95% CI: 60.6–93.4%), PPV 82.1% (95% CI: 63.1–93.9%), and NPV 56.8% (95% CI: 39.5–72.9%). At a CRP threshold of >80 mg/L, the sensitivity was 41.0% (95% CI: 25.6–57.9%), specificity 88.5% (95% CI: 69.8–97.6%), PPV 84.2% (95% CI: 60.4–96.6%), and NPV 50.0% (95% CI: 34.9–65.1%). These higher thresholds increased the specificity and PPV at the expense of sensitivity, providing more clinically actionable information when CRP is markedly elevated.

To determine whether these associations were independent from cardiovascular comorbidities, univariable and multivariable Firth penalized logistic regression models were fitted (Table 4). In the univariable analysis, higher CRP and cardiovascular disease were significantly associated with AFOP. After adjustment for age and cardiovascular disease, CRP remained independently associated with AFOP (adjusted OR = 1.23, 95% CI: 1.08–1.45, *p* = 0.001). In a sensitivity analysis additionally including NLR in the multivariable model, CRP remained independently associated with AFOP (adjusted OR = 1.27, 95% CI: 1.08–1.59, *p* = 0.003), whereas NLR was not independently associated (adjusted OR = 0.92, 95% CI: 0.73–1.13, *p* = 0.457).

### 3.4. Histological Features and IHC Findings

**Figure 2:**
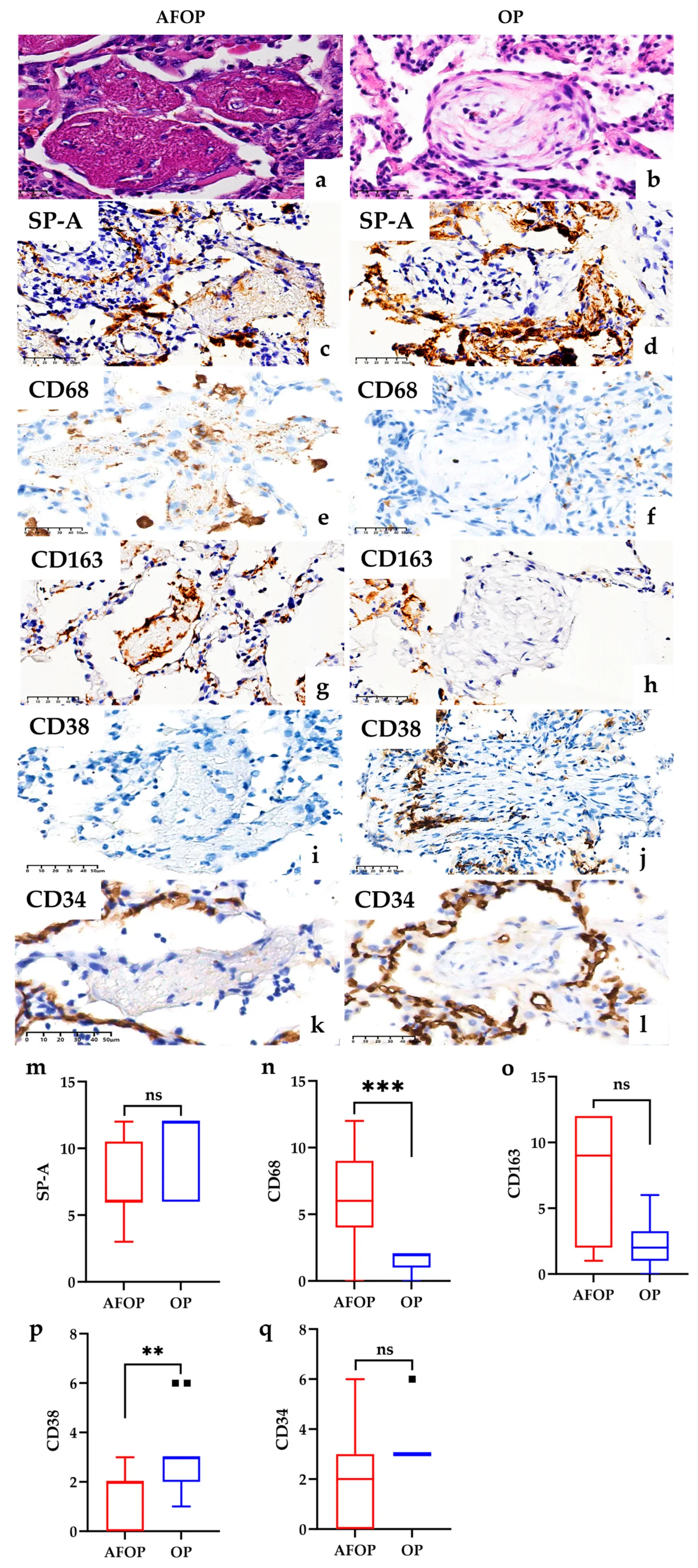
Histological and immunohistochemical features of AFOP and OP. (**a**) Representative histological features of AFOP (H&E staining, ×400). (**b**) Representative histological features of OP (H&E staining, ×400). (**c**,**d**) Immunohistochemical staining for SP-A in AFOP (**c**) and OP (**d**). (**e**,**f**) Immunohistochemical staining for CD68 in AFOP (**e**) and OP (**f**). (**g**,**h**) Immunohistochemical staining for CD163 in AFOP (**g**) and OP (**h**). (**i**,**j**) Immunohistochemical staining for CD38 in AFOP (**i**) and OP (**j**). (**k**,**l**) Immunohistochemical staining for CD34 in AFOP (**k**) and OP (**l**). (**m**–**q**) Semi-quantitative IRS comparing AFOP and OP groups for SP-A (**m**), CD68 (**n**), CD163 (**o**), CD38 (**p**), and CD34 (**q**). Data are presented as median (interquartile range). Statistical significance in (**m**–**q**) is based on BH-adjusted q values; only CD68 and CD38 remained significant after correction. *** q < 0.001, ** q < 0.01; ns, not significant. Box plots show the median (horizontal line), interquartile range (box), whiskers (range within 1.5 IQR), and outliers (black squares). Original magnification: ×300–400 (**c**–**l**). AFOP: acute fibrinous and organizing pneumonia; OP: organizing pneumonia; SP-A: surfactant protein A; IRS: immunoreactivity scores; BH: Benjamini–Hochberg.

The AFOP group was primarily characterized by the presence of intra-alveolar fibrin in the form of “fibrin balls”. Secondary features included mild-to-moderate interstitial infiltration, alveolar septal thickening, type II pneumocyte hyperplasia, focal myxoid fibroblastic tissue, intra-alveolar macrophages, and alveolar edema (Figure 2a). The OP group predominantly showed patchy intraluminal organizing fibrosis in distal airspaces, with mild chronic interstitial inflammation (Figure 2b).

To further characterize the immunophenotypic differences between the two groups, immunohistochemical staining was performed for a panel of markers (detailed scores in Table A3). The interobserver agreement for the histological classification was excellent (Cohen’s kappa = 0.952, *p* < 0.001). For semi-quantitative IRS scores, weighted kappa across the 10 markers with score variability ranged from 0.803 to 0.947, indicating good-to-excellent agreement (Table A4). Vimentin and desmin showed no score variability and were 100% concordant; therefore, kappa could not be calculated for them. Among alveolar epithelial markers, SP-A expression was lower in the AFOP group than in the OP group (6.00 vs. 12.00, *p* = 0.020, q = 0.060) (Figure 2c,d,m), whereas SP-B did not differ significantly between groups (*p* = 0.756). Regarding macrophage-associated markers, the AFOP group exhibited a markedly higher expression of CD68 (6.00 vs. 2.00, *p* < 0.001, q < 0.001) (Figure 2e,f,n) and CD163 (9.00 vs. 2.00, *p* = 0.015, q = 0.060) (Figure 2g,h,o) compared with the OP group. For lymphocyte markers, CD38 expression was significantly lower in the AFOP group (2.00 vs. 3.00, *p* < 0.001, q = 0.005) (Figure 2i,j,p), while CD3 and CD20 showed no significant differences between groups (all *p* > 0.05). The vascular marker CD34 was slightly lower in the AFOP group (2.00 vs. 3.00, *p* = 0.028, q = 0.067) (Figure 2k,l,q), whereas D2-40 did not differ significantly (*p* = 0.317). No significant differences were observed in the expression of mesenchymal markers, including desmin, vimentin, and α-SMA, between the two groups (all *p* > 0.05). After BH correction across all 12 IHC markers, only CD68 and CD38 remained statistically significant. Among the markers that remained significant after correction, CD68 showed a large effect size (Cliff’s delta = 0.85, 95% CI: 0.29–0.98), indicating higher expression in AFOP, while CD38 also demonstrated a large effect size (Cliff’s delta = −0.65, 95% CI: −0.85–−0.38), consistent with lower expression in AFOP.

### 3.5. Treatment and Clinical Outcomes

The treatment and clinical outcomes of the two groups are summarized in Table 5. The majority of patients in both groups received antibiotics alone (AFOP 60.42% vs. OP 63.64%), and the overall distribution of treatment regimens did not differ significantly between groups (*p* = 0.941). Among the 10 AFOP patients and 7 OP patients who received glucocorticoids, the initial prednisone-equivalent dose and treatment duration were comparable (both *p* > 0.05).

Oxygen therapy was received by 71.43% of AFOP patients and 55.56% of OP patients (*p* = 0.385). Among all patients who received oxygen therapy, four required non-invasive or invasive mechanical ventilation, all of whom belonged to the AFOP group. The median duration of hypoxemia (*p* = 0.847) and length of hospital stay (*p* = 0.142) did not differ significantly between groups. The majority of patients in both groups achieved partial or complete clinical improvement (95.92% vs. 100.00%; *p* = 0.322). During follow-up, relapse occurred in two AFOP patients (4.08%) and one OP patient (2.78%) (*p* > 0.999). Three patients died, all in the AFOP group, although this difference did not reach statistical significance (*p* = 0.281).

Given the very low event rates, the post hoc power was only 7.6% for mortality and 20.6% for mechanical ventilation. These comparisons were exploratory, and the absence of statistical significance should not be interpreted as evidence of no true difference.

## 4. Discussion

This study systematically compared 49 patients with AFOP and 36 patients with OP across multiple dimensions. Our findings demonstrate that, despite largely overlapping clinical and radiological presentations, AFOP and OP diverge significantly in systemic inflammatory responses and pulmonary immunophenotypes. AFOP is characterized by more intense inflammation and a macrophage-predominant pattern.

The two groups were similar in age, sex, smoking history, clinical presentations, and radiological patterns. Comorbidities were also largely comparable between the two groups, with the exception of a higher prevalence of cardiovascular disease in AFOP.

Despite their clinical–radiological similarities, AFOP and OP differed substantially in the magnitude of systemic inflammation. In our study, patients with AFOP exhibited significantly higher CRP levels and NLR compared with those with OP. This finding is consistent with and extends prior observations. Nagata et al. reported that even focal intra-alveolar fibrin in otherwise typical OP was independently associated with markedly elevated CRP and predicted early relapse [11]. Onishi et al. demonstrated that patients with a dominant AFOP pattern had a significantly higher serum CRP than those with OP lacking fibrin deposition. They proposed that AFOP may represent an early hyperacute phase shared by various inflammatory lung diseases [10]. Our direct comparison not only corroborates this CRP disparity but also demonstrates moderate discriminatory value, with the CRP yielding an AUC of 0.798 for distinguishing AFOP from OP. The concomitant elevation of NLR in AFOP suggests that this systemic inflammatory response is characterized by innate immune predominance, a pattern typically associated with more severe acute tissue injury.

After adjustment for age and cardiovascular disease, CRP remained independently associated with AFOP (adjusted OR per 10 mg/L = 1.23, 95% CI: 1.08–1.45). This suggests that the elevated CRP in AFOP is not solely attributable to cardiovascular comorbidity. Cardiovascular disease also showed an independent association, although the extremely wide CI reflects the small number of events and limits interpretation. Of note, the Youden-derived optimal CRP cut-off was only mildly elevated. When higher thresholds were evaluated, the specificity and PPV increased, albeit with reduced sensitivity. Thus, marked CRP elevation provides stronger supportive evidence for AFOP, although these thresholds still require external validation in independent cohorts. However, because the CRP was missing in 20/85 patients, the cut-off and AUC should be interpreted as exploratory.

Beyond the systemic inflammatory response, the two groups also diverged at the tissue level. Immunohistochemical analysis of alveolar epithelial, macrophage, lymphocyte, vascular, and mesenchymal markers revealed distinct immunophenotypic profiles. Among the alveolar epithelial markers, SP-A expression was lower in AFOP than in OP in the uncorrected analysis, but this difference was not significant after multiple comparison correction and should be interpreted as exploratory. SP-B did not differ between groups. SP-A is a lung collectin that plays critical roles in surfactant homeostasis and local innate immune regulation within the distal airspaces [15]. To date, SP-A has been investigated in AFOP almost exclusively at the serum level, where it is reportedly elevated [16]. These findings raise the possibility of tissue-level SP-A downregulation in AFOP, but this requires confirmation. Notably, SP-A levels in bronchoalveolar lavage fluid are also reduced in other diffuse parenchymal lung diseases, including OP, compared with healthy controls [17]. This reduction is attributed to decreased surfactant production and increased leakage into the systemic circulation. Whether a similar mechanism operates in AFOP, or whether it could account for the apparent SP-A difference observed in this study, remains to be determined.

The most pronounced immunophenotypic difference was observed in macrophage-associated markers. The CD68 expression was significantly higher in AFOP than in OP. CD163 also tended to be higher in the uncorrected analysis, but this difference did not survive multiple comparison correction. Notably, the distribution of macrophages differed between groups: in AFOP, CD68+ and CD163+ cells were also present within intra-alveolar fibrin deposits, whereas in OP, they were predominantly interstitial. The presence of macrophages within AFOP lesions has been documented in case reports. Santos et al. described CD68+ macrophages surrounding fibrin balls [6], and Taira et al. reported abundant CD163+ macrophages within fibrin balls and alveolar spaces [18]. Macrophages are also observed in OP, where they participate in the clearance of intra-alveolar exudates [19,20]. However, no prior study has systematically compared the macrophage density or distribution between AFOP and OP. Our data therefore provide direct evidence that the CD68+ macrophage density is significantly higher in AFOP, with a similar trend for CD163 that requires confirmation. Moreover, these cells were more extensively distributed in AFOP, including within intra-alveolar fibrin deposits.

In contrast to the macrophage markers, CD38, a marker of plasma cells and activated lymphocytes, was significantly lower in AFOP than in OP. Together with comparable CD3 and CD20 levels between groups, this suggests a relatively muted adaptive immune response in AFOP. This aligns with the innate immune predominance suggested by elevated NLR and robust macrophage infiltration in AFOP. Although the number of markers surviving correction was limited, the overall pattern, including trends for CD163 and NLR, is consistent with an innate immune-predominant inflammation that requires further confirmation.

The vascular marker CD34 also revealed notable differences. Consistent with previous studies [19,21,22], CD34+ microvessels in OP were readily identified in the interstitium and within Masson bodies. In contrast, CD34+ microvessels were scarce or absent within fibrin balls. Although the semi-quantitative CD34 score was lower in AFOP in uncorrected analysis, this difference did not survive correction. Therefore, the apparent scarcity of microvessels within fibrin balls compared with Masson bodies should be regarded as a qualitative histological observation. This divergence, the incorporation of microvessels into Masson bodies but not into fibrin balls, may reflect different tissue repair patterns. Mesenchymal markers, including desmin, vimentin, and α-SMA, did not differ between the two groups, suggesting that myofibroblastic activation is not enhanced in AFOP.

Notably, all three deaths and all cases requiring mechanical ventilation occurred in the AFOP group, whereas all OP patients achieved clinical improvement without mechanical ventilation. Given the low event rates and limited power, these comparisons of outcomes were exploratory and should not be overinterpreted. Nevertheless, they are consistent with previous reports of higher mortality and mechanical ventilation rates in AFOP [4,14,23], as well as the generally favorable prognosis of OP [2,9].

Our study has some limitations. First, its retrospective design and modest sample size limited the statistical power to detect differences in infrequent outcomes such as mortality and mechanical ventilation, and missing data were handled by complete-case analysis without imputation, which may introduce bias. Second, semi-quantitative IHC scoring is inherently less precise than digital quantification, and digital image analysis was not performed, although all assessments were independently performed by two experienced pathologists. Third, most patients underwent PLB; therefore, the influence of the biopsy type on the diagnostic accuracy could not be formally assessed. Finally, the cross-sectional nature of this study precludes any conclusions regarding the longitudinal relationship between AFOP and OP, and the immunophenotypic interpretations remain descriptive and require validation by flow cytometry or transcriptomic profiling.

## 5. Conclusions

In conclusion, despite clinical and radiological overlap, AFOP and OP showed distinct systemic inflammatory and pulmonary immunophenotypic profiles. AFOP was associated with a more intense acute-phase inflammatory response and a macrophage-predominant tissue pattern, although the immunophenotypic interpretations remain descriptive and require validation by flow cytometry or transcriptomic profiling. AFOP also showed qualitatively reduced CD34+ microvessels within fibrin deposits, a finding that requires confirmation by quantitative vascular assessment. The longitudinal relationship between AFOP and OP remains to be clarified.

## Figures and Tables

**Table 1:** Clinical characteristics of patients with AFOP and OP.

	AFOP (*n* = 49)	OP (*n* = 36)	*p* Value
Age, y	54.34 ± 15.15	53.76 ± 14.82	0.318 ^a^
Sex, *n* (%)			0.691 ^b^
Male	32 (65.31)	22 (61.11)	
Female	17 (34.69)	14 (38.89)	
BMI, kg/m^2^	21.58 ± 3.31	22.33 ± 3.16	0.370 ^a^
Smoking status, *n* (%)			0.400 ^b^
Former/Current	21 (42.86)	10 (33.33)	
Never	28 (57.14)	20 (66.67)	
Clinical symptoms *, *n* (%)			
Fever	14 (28.57)	15 (41.67)	0.208 ^b^
Dyspnea	8 (16.33)	5 (13.89)	0.758 ^b^
Cough	32 (65.31)	24 (66.67)	0.896 ^b^
Chest pain	14 (28.57)	7 (19.44)	0.335 ^b^
Sputum	19 (38.78)	17 (47.22)	0.436 ^b^
Underlying diseases *, *n* (%)			
None	5 (10.20)	5 (15.15)	0.513 ^b^
Respiratory system diseases	22 (44.90)	10 (30.30)	0.184 ^b^
Neoplastic diseases	15 (30.61)	7 (21.21)	0.346 ^b^
Hematological diseases	1 (2.04)	1 (3.03)	>0.999 ^c^
Cardiovascular system diseases	11 (22.45)	1 (3.03)	**0.023 ^c^**
Urinary system diseases	6 (12.24)	0 (0.00)	0.076 ^c^
Metabolic diseases	11 (22.45)	3 (9.09)	0.115 ^c^
Digestive system disease	15 (30.61)	7 (21.21)	0.346 ^b^
Autoimmune diseases	5 (10.20)	0 (0.00)	0.079 ^c^
Biopsy approach, *n* (%)			0.156 ^c^
PLB	47 (95.92)	30 (83.33)	
TBLB	2 (4.08)	6 (16.67)	

Data are presented as mean ± standard deviation or *n* (%). Missing data: smoking status (OP, *n* = 6) and underlying diseases (OP, *n* = 3); percentages are based on available cases. * Some patients had more than one clinical symptom or underlying disease. ^a^ T-test; ^b^ Chi-square test; ^c^ Fisher’s exact test. AFOP: acute fibrinous and organizing pneumonia; OP: organizing pneumonia; BMI: body mass index; PLB: percutaneous lung biopsy; TBLB: transbronchial lung biopsy.

**Table 2:** Radiological characteristics of AFOP and OP patients.

	AFOP (*n* = 49)	OP (*n* = 36)	*p* Value
Dominant pattern, *n* (%)			0.712 ^a^
GGO/consolidation-dominant	19 (38.78)	13 (36.11)	
Nodule-dominant	25 (51.02)	17 (47.22)	
Fibrosis-dominant	5 (10.20)	6 (16.67)	
Location, *n* (%)			0.275 ^a^
Upper lobe-predominant	12 (24.49)	4 (11.11)	
Lower lobe-predominant	12 (24.49)	9 (25.00)	
Diffuse/random	25 (51.02)	23 (63.89)	
Specific radiological findings *, *n* (%)			
Nodule or mass	39 (79.59)	27 (75.00)	0.616 ^a^
Consolidation	20 (40.82)	11 (30.56)	0.332 ^a^
Ground glass opacities	16 (32.65)	7 (19.44)	0.176 ^a^
Fibrosis	25 (51.02)	18 (50.00)	0.926 ^a^
Pleural effusion	9 (18.37)	11 (30.56)	0.191 ^a^
Lymphadenopathy	23 (46.94)	13 (36.11)	0.318 ^a^
Reverse halo sign	0 (0.00)	1 (2.78)	0.424 ^b^
Air bronchograms	2 (4.08)	1 (2.78)	>0.999 ^b^

Data are presented as *n* (%). * Some patients had more than one radiological change. ^a^ Chi-square test; ^b^ Fisher’s exact test. AFOP: acute fibrinous and organizing pneumonia; OP: organizing pneumonia; GGO: ground glass opacities.

**Table 3:** Laboratory findings of AFOP and OP patients.

	AFOP (*n* = 49)	OP (*n* = 36)	*p* Value *
WBC, ×10^9^/L	8.68 (6.21–11.95)	7.95 (5.08–9.52)	0.125
CRP, mg/L	80.45 (43.23–146.25)	12.65 (2.94–26.95)	**<0.001**
ESR, mm/h	87.50 (62.75–120.00)	67.50 (26.75–103.50)	0.084
PCT, ng/mL	0.08 (0.04–0.13)	0.06 (0.03–0.09)	0.331
SII, ×10^9^/L	1322.31 (789.09–1631.60)	786.82 (185.24–1157.38)	0.111
NLR	4.56 (3.23–6.10)	2.42 (1.01–3.28)	**0.013**

Data are presented as median (interquartile range). Analyses for each marker were based on available cases (AFOP/OP): WBC (31/26), CRP (39/26), ESR (20/17), PCT (19/12), SII (49/34), NLR (49/34). * Mann–Whitney U test. AFOP: acute fibrinous and organizing pneumonia; OP: organizing pneumonia; WBC: white blood cell; CRP: C-reactive protein; ESR: erythrocyte sedimentation rate; PCT: procalcitonin; SII: systemic immune-inflammation index; NLR: neutrophil-to-lymphocyte ratio.

**Table 4:** Logistic regression analysis of factors associated with AFOP versus OP.

Variables	Univariable		Multivariable	
	OR (95%CI)	*p* Value	OR (95%CI)	*p* Value
Age	0.99 (0.96–1.01)	0.326	0.98 (0.94–1.02)	0.298
CRP	1.23 (1.08–1.44)	**<0.001**	1.23 (1.08–1.45)	**0.001**
NLR	1.09 (0.99–1.25)	0.074	–	–
Cardiovascular system diseases	6.47 (1.42–61.90)	**0.013**	24.20 (2.32–3376.34)	**0.004**

Univariable and multivariable logistic regression analyses used Firth penalized logistic regression to account for sparse events and potential separation. Multivariable models were adjusted for age and cardiovascular disease; CRP per 10 mg/L. In a sensitivity analysis additionally including NLR, the adjusted OR for CRP was 1.27 (95% CI 1.08–1.59, *p* = 0.003) and for NLR was 0.92 (95% CI 0.73–1.13, *p* = 0.457). OR: odds ratio; CI: confidence interval; CRP: C-reactive protein; NLR: neutrophil-to-lymphocyte ratio.

**Table 5:** Treatment and clinical outcomes of AFOP and OP patients.

	AFOP (*n* = 49)	OP (*n* = 36)	*p* Value
Treatment, *n* (%)			0.941 ^a^
Antibiotics only	29 (60.42)	21 (63.64)	
Steroids only	1 (2.08)	0 (0.00)	
Steroids and antibiotics	9 (18.75)	7 (21.21)	
Others	9 (18.75)	5 (15.15)	
Glucocorticoid initial dose, mg/day	30.00 (12.50–50.00)	45.00 (18.35–50.00)	0.960 ^b^
Glucocorticoid duration, days	21.00 (10.75–64.25)	18.00 (11.50–389.50)	0.515 ^b^
Oxygen therapy, *n* (%)	35 (71.43)	20 (55.56)	0.385 ^a^
Nasal cannula	29 (82.86)	20 (100.00)	
Mask	2 (5.71)	0 (0.00)	
Non-invasive ventilation	3 (8.57)	0 (0.00)	
Invasive ventilation	1 (2.86)	0 (0.00)	
Duration of hypoxemia, days	11.00 (3.00–23.50)	11.00 (6.00–16.50)	0.847 ^b^
Hospital stay, days	24.00 (15.75–32.25)	14.00 (11.50–36.50)	0.142 ^b^
Treatment response, *n* (%)			0.322 ^a^
Complete clinical improvement	14 (28.57)	6 (16.67)	
Partial clinical improvement	33 (67.35)	30 (83.33)	
No clinical improvement	2 (4.08)	0 (0.00)	
Clinical outcomes, *n* (%)			
Relapse	2 (4.08)	1 (2.78)	>0.999 ^a^
Death	3 (6.12)	0 (0.00)	0.281 ^a^
Follow-up time, days	1481.00 (175.00–1961.75)	1559.50 (62.75–2084.50)	>0.999 ^b^

Data are presented as median (interquartile range) or *n* (%). Treatment data missing for one AFOP and three OP patients; glucocorticoid data only for patients receiving steroids (AFOP *n* = 10, OP *n* = 7). Glucocorticoid dose expressed as prednisone equivalent. ^a^ Fisher’s exact test; ^b^ Mann–Whitney U test. AFOP: acute fibrinous and organizing pneumonia; OP: organizing pneumonia.

## Data Availability

All relevant data are available from the corresponding author on reasonable request.

## Abbreviations

The following abbreviations are used in this manuscript:

OP	Organizing pneumonia
AFOP	Acute fibrinous and organizing pneumonia
CRP	C-reactive protein
NLR	Neutrophil-to-lymphocyte ratio
PLB	Percutaneous lung biopsy
TBLB	Transbronchial lung biopsy
FFPE	Formalin-fixed paraffin-embedded
H&E	Hematoxylin and eosin
IHC	Immunohistochemistry
SP-A	Surfactant protein A
SP-B	Surfactant protein B
α-SMA	α-smooth muscle actin
IRS	Immunoreactivity score
CI	Confidence interval
BH	Benjamini–Hochberg
ROC	Receiver operating characteristic
PPV	Positive predictive value
NPV	Negative predictive value
LR	Likelihood ratios
AUC	Area under the curve
BMI	Body mass index
GGO	Ground-glass opacity
WBC	White blood cell count
ESR	Erythrocyte sedimentation rate
PCT	Procalcitonin
SII	Systemic immune-inflammation index
OR	Odds ratio

## Appendix A

**Table A1:** Histological diagnostic criteria for AFOP and OP groups.

Group	Histological Criteria
AFOP	Primary histological features: (i) intra-alveolar fibrin in the form of “fibrin balls”; Secondary histological features:(i) mild to moderate acute or chronic interstitial infiltration; (ii) thickening of alveolar septa; (iii) hyperplasia of type II pneumocytes; (iv) minimal or focal presence of myxoid fibroblastic tissue in the alveolar walls; (v) presence of macrophages within the alveolar spaces; and (vi) edema of alveoli.
OP	(i) Intralumenal organizing fibrosis in distal airspaces distributed in a patchy pattern; (ii) Mild interstitial chronic inflammation.

AFOP: acute fibrinous and organizing pneumonia; OP: organizing pneumonia.

**Table A2:** Detailed information of antibodies used in IHC.

Antibody Name	Clone Number	Vendor	Dilution
Surfactant protein A (SP-A)	6F10	Gene Tech (Shanghai) Co., Ltd., Shanghai, China	1/800
Surfactant protein B (SP-B)	1B9	ZSGB-BIO, Beijing, China	1/100
CD34	EP88	ZSGB-BIO, Beijing, China	1/200
D2-40	D2-40	ZSGB-BIO, Beijing, China	1/200
Vimentin	UMAB159	ZSGB-BIO, Beijing, China	1/100
Desmin	MX046	Maixin Biotech, Fuzhou, China	1/200
α-smooth muscle actin (α-SMA)	UMAB237	ZSGB-BIO, Beijing, China	1/200
CD68	PG-M1	ZSGB-BIO, Beijing, China	Ready-to-use
CD163	10D6	ZSGB-BIO, Beijing, China	1/100
CD3	LN10	ZSGB-BIO, Beijing, China	1/100
CD20	L26	Ventana Medical Systems, Tucson, AZ, USA	Ready-to-use
CD38	MX044	ZSGB-BIO, Beijing, China	1/200

**Table A3:** Detailed IHC scores of AFOP and OP patients.

	AFOP (*n* = 49)	OP (*n* = 36)	*p* Value *	BH q Value ^#^
SP-A	6.00 (6.00–10.50)	12.00 (6.00–12.00)	**0.020**	0.060
SP-B	4.00 (3.00–9.00)	4.00 (2.00–6.00)	0.756	0.907
CD68	6.00 (4.00–9.00)	2.00 (1.00–2.00)	**<0.001**	**<0.001**
CD163	9.00 (2.00–12.00)	2.00 (1.00–3.25)	**0.015**	0.060
Desmin	3.00 (3.00–3.00)	3.00 (3.00–3.00)	>0.999	>0.999
Vimentin	12.00 (12.00–12.00)	12.00 (12.00–12.00)	>0.999	>0.999
α-SMA	3.00 (3.00–3.00)	3.00 (3.00–6.00)	0.219	0.375
CD34	2.00 (0.00–3.00)	3.00 (3.00–3.00)	**0.028**	0.067
D2-40	2.00 (2.00–3.00)	1.00 (0.00–3.00)	0.317	0.476
CD3	3.00 (3.00–6.00)	4.00 (3.00–6.00)	0.883	0.963
CD20	3.00 (3.00–3.00)	3.00 (3.00–3.00)	0.065	0.130
CD38	2.00 (0.00–2.00)	3.00 (2.00–3.00)	**<0.001**	**0.005**

Data are presented as median (interquartile range). * Mann–Whitney U test. ^#^ Benjamini–Hochberg correction: false discovery rate-adjusted *p*-value across the 12 IHC markers; *p* < 0.05 was considered significant for uncorrected analyses, and q < 0.05 was considered significant after Benjamini–Hochberg correction. For desmin and vimentin, all values were identical between groups; thus, *p* > 0.999 and q > 0.999. IHC: immunohistochemistry; AFOP: acute fibrinous and organizing pneumonia; OP: organizing pneumonia; SP-A: surfactant protein A; SP-B: surfactant protein B; α-SMA: α-smooth muscle actin.

**Table A4:** Interobserver agreement for IHC markers.

Marker	Weighted Kappa
SP-A	0.899
SP-B	0.861
CD34	0.892
D2-40	0.921
CD68	0.916
Desmin	NC
Vimentin	NC
α-SMA	0.911
CD3	0.947
CD20	0.882
CD38	0.907
CD163	0.803

Weighted kappa was calculated with quadratic weights. NC: not calculable due to zero variance; both raters assigned identical scores in all cases, representing 100% concordance. SP-A: surfactant protein A; SP-B: surfactant protein B; α-SMA: α-smooth muscle actin.
